# Mutations in ERG11, TAC1B, and CDR1 reduce fluconazole accumulation in drug-resistant Candidozyma auris isolates

**DOI:** 10.1128/mbio.03957-25

**Published:** 2026-02-06

**Authors:** Brooke D. Esquivel, Amanda Santos, Jeffrey M. Rybak, Darian J. Santana, P. David Rogers, Theodore C. White

**Affiliations:** 1 Division of Biological and Biomedical Systems, School of Science and Engineering, University of Missouri-Kansas City 318268, Kansas City, Missouri, USA; 2 Mycotic Diseases Branch, NCEZID, Centers for Disease Control and Prevention 1242, Atlanta, Georgia, USA; 3 Department of Pharmacy and Pharmaceutical Sciences, St. Jude Children's Research Hospital 5417, Memphis, Tennessee, USA; Universiteit Gent, Gent, Belgium

**Keywords:** drug accumulation, C. auris, drug import, drug efflux, mechanisms of resistance, fluconazole

## Abstract

Fluconazole (FLC)-resistant Candidozyma auris isolates have reduced drug accumulation compared to azole-susceptible isolates. Of 119 C. auris isolates, 83 out of 87 resistant isolates (~95%) had extremely low fluconazole uptake, whereas 30 out of 32 susceptible isolates (~93%) had high fluconazole uptake. In search of a genetic explanation for this phenomenon, we compared metadata for TAC1B and CDR1 single-nucleotide polymorphisms (SNPs) and found overlap with many but not all isolates that are FLC resistant. We found that CDR1 mutations are common in resistant isolates from Clade 1, and TAC1B mutations are commonly found in resistant isolates from clades 1 and 3. There is clearly an association between FLC resistance and certain CDR1 and TAC1B polymorphisms, but mutations in these genes do not account for all mechanisms of resistance in this species and do not account for the difference in FLC accumulation. However, when ERG11 SNPs were included in the analysis, there is a clear correlation between low FLC accumulation and isolates that have one of five ERG11 variants and also high FLC accumulation and isolates that have non-variant ERG11 sequences. The ERG11 mutations F126L, K143R, V125/F126L, Y132F, or Y501H are correlated to fluconazole resistance and reduced fluconazole accumulation. This is a unique characteristic of C. auris, suggesting mutations in ERG11 can cause changes in the ergosterol biosynthesis pathway and membrane composition, organization, and permeability.

**IMPORTANCE:**

Candidozyma auris is a global human health threat because of its near-universal resistance to the antifungal fluconazole as well as a predisposition to multidrug resistance among clinical isolates. The underlying mechanisms of antifungal drug resistance in this species are still largely under investigation, and these efforts are significantly supported by research that increase our understanding of unique aspects of C. auris biology. We have identified a correlation between C. auris isolates’ susceptibility to fluconazole and intracellular drug accumulation in which drug-resistant isolates have significantly reduced intracellular fluconazole compared to isolates that are susceptible to fluconazole. We have proposed a mechanism for this phenomenon and demonstrated important roles for mutations in ERG11, TAC1B, and CDR1 gene sequences for drug resistance.

## INTRODUCTION

Candidozyma auris (previously classified as Candida auris) was named a priority pathogen in the Critical Priority Group by the World Health Organization (WHO) in 2022. The Centers for Disease Control and Prevention (CDC) named C. auris an urgent threat because of the intrinsic single and multidrug resistance predisposition in many isolates, as well as a rapid rise in cases over the last few years (1). There were over 4,514 clinical cases of C. auris infections in the United States in 2023, and many more patients were found to be colonized, with cases continuing to rise (1). C. auris is most frequently isolated from the medical environment, where this pathogen is known to be transmitted horizontally between patients and surfaces and can cause deadly systemic disease, although it is also notable that C. auris is increasingly being found in other environments and has also been isolated from dogs, fruits and crops, and wastewater around the world (2–6).

Echinocandins are the recommended first-line treatment for most infections with Candida species (7); however, the azole class of drugs (8) remains the standard of care in many health care centers and is commonly relied upon for prophylaxis of Candida infections among vulnerable patient populations (7, 8). Azoles inhibit ergosterol biosynthesis by interfering with a bottle-neck enzyme along the pathway (Erg11 in Candida), which converts lanosterol to an ergosterol precursor (9). Ergosterol is the primary sterol in the fungal cell membrane and plays a major role in maintaining plasma membrane integrity and function (8). There are no established azole susceptibility breakpoints established specifically for C. auris, and so guidelines for defining azole resistance and susceptibility are based on the current clinical experience, limited pharmacokinetic and pharmacodynamic data, and breakpoints established for closely related species such as C. albicans (1). C. auris isolates have a near-universal resistance to the azole fluconazole (FLC) and a wide range of minimum inhibitory concentrations (MICs) to other antifungals (10). Fluconazole resistance in C. auris is tentatively defined as having an MIC of ≥32 µg/mL (1). In the United States, about 90% of C. auris isolates have been determined to be resistant to fluconazole (1).

The fungistatic nature of azole antifungal drugs allows the potential for fungal adaptation and evolution of resistance mechanisms to this class of drugs (11). Similar resistance mechanisms arise independently in the majority of fungal pathogens and continue to be studied in order to better understand failed treatments and inform on possible treatment alternatives and new drug development (12). The increased efflux of antibiotic drugs out of the cell, through the upregulation of drug efflux pumps, is a core mechanism of drug resistance in many fungal pathogens (13) and can be a determining factor for treatment outcomes. Although the transport mechanisms for many drugs remain to be identified, it has been demonstrated that differences in the expression level of efflux transporters contribute to variability in fungal susceptibility and drug effectiveness (14). Several families of efflux systems capable of antifungal drug efflux have been described, such as ATP-binding cassette (ABC) and major facilitator superfamily (MFS) transporters (13, 14).

Because ergosterol is the major sterol component of fungal membranes and the primary fungal component affected by treatment with azoles, a change to ERG11 is another acquired resistance mechanism found in many Candida species (11, 13). This occurs independent of, or in addition to, changes in transporter expression and function. Increased ergosterol production and an altered sterol composition in the plasma membrane caused by upregulation of the ERG11 gene are distinct fungal adaptations to azole treatment (13, 15).

A related ERG11 and azole-specific resistance strategy is the presence of point mutations in ERG11, which may alter the amino acid composition and tertiary structure of the enzyme. Structural changes, especially near the active site of the enzyme, can result in decreased binding affinity of the azoles to Erg11p and thus reduced susceptibility to azoles in those strains (9). Some studies have identified point mutations in ERG11 in azole-resistant fungal isolates, often developed as a result of long-term exposure to antifungals (16, 17). Over 160 Erg11 amino acid substitutions have been reported in pathogenic fungi; however, not all of them are directly associated with FLC resistance (18). This suggests that the Erg11 enzyme is prone to, but flexible with, different structural changes.

Perhaps the most rigorously studied Candida ERG11 mutations are those found in C. albicans. Studies in C. albicans have defined three regions of amino acid residues, which are particularly permissive/susceptible to amino acid substitutions and could correspond to conformational changes in the protein. These regions include residues 105–165, 266–287, and 405–488 (9). In C. auris, there are also 3 hot spot mutations that have been identified in Erg11 of fluconazole-resistant isolates (Y132F, K143R, and VF125AL [sometimes referred to simply as F126L]) (19). We have previously shown that each of these mutations significantly increases C. auris resistance to fluconazole and the structurally similar voriconazole (VORI) but has a more modest impact on susceptibility to later-generation azoles such as posaconazole (POSA).

Comparative genomic analyses indicate that the C. auris genome contains conserved genes within the CTG clade that are associated with virulence and antifungal resistance as well as C. auris-specific drug resistance mechanisms. For example, genes encoding the MFS transporter Mdr1, the ABC transporter Cdr1, and the transcriptional regulator Upc2 are present in the C. auris genome, whereas mechanisms of resistance associated with mutations in the transcription factor Tac1B, thought to regulate genes involved in efflux, are unique to C. auris (20). Many genes in the ergosterol biosynthesis pathway are also conserved in C. auris, including ERG11, although the pathway itself has not been fully characterized, and there seems to be some differences compared to related species (5, 20, 21). Previous reports from our labs and others demonstrate contributions of CDR1 and ERG11 for azole drug resistance in C. auris, consistent with resistance mechanisms in other fungal pathogens, as well as mechanisms novel to C. auris such as those associated with Tac1B (19, 20).

In other Candida species, several azole resistance mechanisms often act in concert, which may lead to increases in the MIC, depending on the combination of mutations, expression of efflux pumps, and target gene upregulation. It is reasonable to assume the same is true for C. auris.

We have measured and compared intracellular fluconazole accumulation in over 100 clinical isolates of C. auris that had varying degrees of fluconazole resistance that come from the four predominating genetic clades (named clades I, II, III, and IV). We find a striking correlation between fluconazole resistance and intracellular fluconazole uptake, with resistant isolates accumulating very little fluconazole and susceptible isolates accumulating significantly higher levels of the drug. We find that fluconazole accumulation is affected by TAC1B mutations and CDR1 expression, but the main determinant of fluconazole accumulation is the presence or absence of ERG11 gene mutations.

## RESULTS

**Fig 1:**
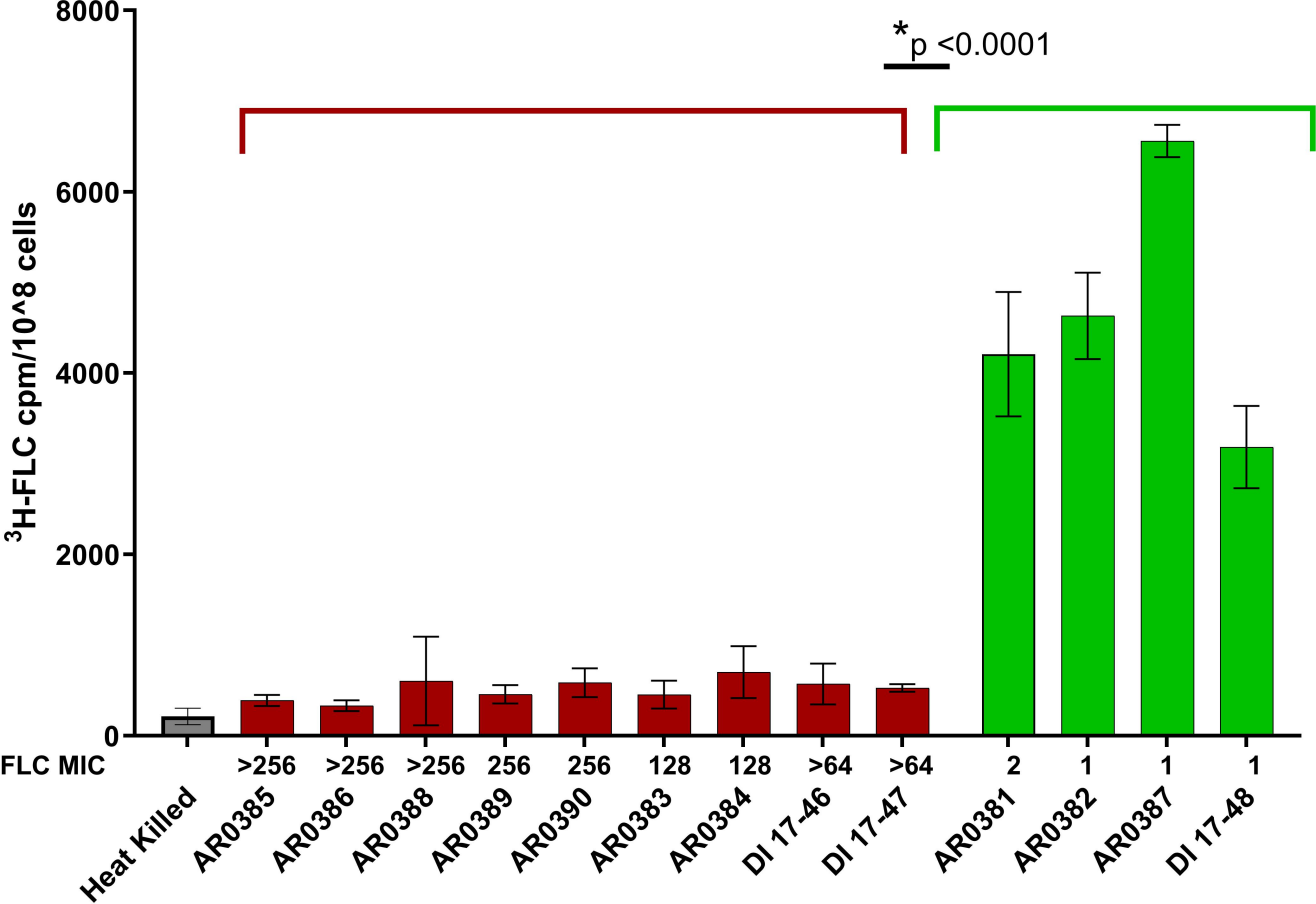
^3^H-FLC accumulation after 24 h in AR Isolate Bank C. auris isolates. Isolates resistant to FLC (red bars) have reduced intracellular FLC accumulation, while isolates susceptible to FLC (green bars) have significantly higher (*P* < 0.0001) intracellular FLC accumulation. Heat-killed cells (gray bar) were used as a control for nonspecific ^3^H-FLC binding. Y axis is a measure of FLC accumulation. X axis includes the isolate name and MICs to FLC.

We obtained a panel of 13 C. auris isolates from the CDC’s Antimicrobial Resistance (AR) Isolate Bank (22) including isolates coming from each clade and with a range of drug susceptibilities (Table 1). We measured intracellular ^3^H-FLC accumulation after treatment in this panel of 13 C. auris isolates. Figure 1 displays the FLC accumulation for each isolate arranged from the highest FLC MIC to lowest (>256–1 µg/mL). We found a striking contrast in fluconazole uptake between the isolates that are fluconazole susceptible (≤16 µg/mL) and resistant (MIC ≥32 µg/mL). The 9 isolates that had FLC MICs above 64 µg/mL (shaded in red) accumulate significantly reduced (*P* < 0.0001) radiolabeled fluconazole, compared to the 4 isolates with FLC MICs at 2 µg/mL and below (shaded in green). Heat-killed cells were used as a control for nonspecific ^3^H-FLC binding and background signal. Heat-killed samples showed minimal accumulation. An example is given in Fig. 1 and not included in subsequent graphs.

**TABLE 1:** *C. auris* AR isolate drug susceptibility profiles^*a*^

Name	AR0381	AR0383	AR0384	DI17-48	AR0385	AR0386	AR0382	AR0387	AR0388	AR0389	AR0390	DI17-46	DI17-47
Clade	2 East Asian	3 South African	3 South African	4 South American	4 South American	4 South American	1 South Asian	1 South Asian	1 South Asian	1 South Asian	1 South Asian	1 South Asian	1 South Asian
FLU	2	128	128	1	>256	>256	1	1	>256	256	256	>64	>64
VORI	0.03	4	1	<0.06	16	16	0.5	0.03	2	4	0.5	1	2
ITRA	0.125	0.5	1	0.5	1	0.5	1	0.25	0.5	0.25	8	1	1
POSA	0.06	0.5	0.5	0.25	1	0.5	0.5	0.002	0.25	0.125	0.03	0.25	0.25
AMB	0.38	0.38	0.5	1	0.5	0.5	0.38	0.75	1.5	4	4	2	1
ANI	0.25	1	2	NA^*b*^	1	1	0.25	0.5	0.5	1	1	NA	NA
CAS	0.125	0.25	16	0.125	0.5	0.5	0.5	0.25	1	0.5	0.5	0.25	0.25
MIC	0.125	1	2	0.06	0.5	0.25	0.25	0.5	0.125	0.25	0.25	0.125	0.25
FCY	2	0.5	0.5	NA	0.5	0.5	0.125	8	0.125	128	128	NA	NA

^*a*^ MIC values for each drug are reported as µg/mL.

^*b*^ NA, test not performed.

**Fig 2:**
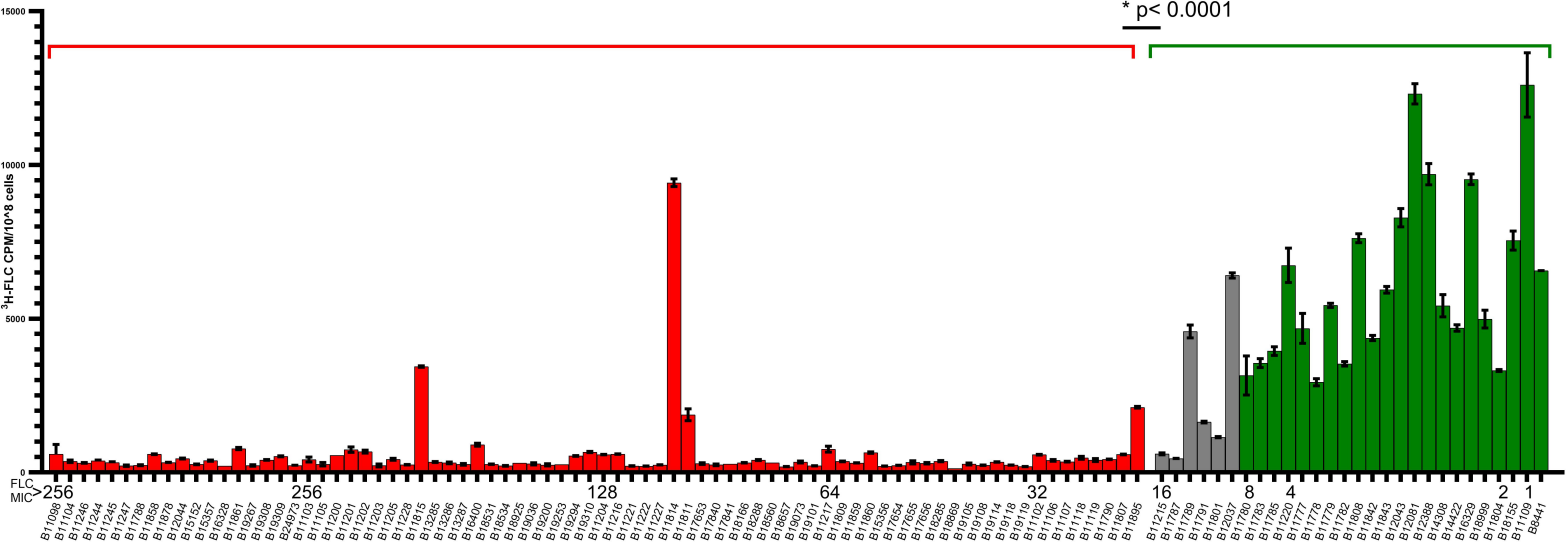
^3^H-FLC accumulation at 24 h in expanded isolate collection. Isolates are sorted from high FLC MIC to low FLC MIC. Red bars indicate FLC MIC >16 µg/mL. Gray bars indicate FLC MIC = 16 µg/mL. Green bars indicate FLC MIC <16 µg/mL. Y axis is a measure of FLC accumulation. X axis includes the isolate name and FLC MIC. Resistant isolates (red bars) have significantly reduced (*P* < 0.0001) FLC accumulation compared to the susceptible isolates (gray and green bars).

To expand the intracellular drug accumulation analysis to include more isolates, we obtained 105 C. auris isolates from the CDC collection (and B12037 from Philippe Dufresne) and measured the intracellular accumulation of ^3^H-FLC in these isolates. Figure 2 shows the isolates sorted from high to low FLC MIC (>256–2 µg/mL). The correlation between FLC-resistant isolates and reduced FLC accumulation remained consistent with the data from the additional isolates. The expanded analysis showed that isolates with FLC MIC above 16 µg/mL (red bars) showed significantly reduced (*P* < 0.0001) FLC accumulation compared to FLC-susceptible isolates. Isolates with an FLC MIC of <16 µg/mL (green bars) have high FLC accumulation. Isolates with an FLC MIC of 16 µg/mL (gray bars) showed some variability in FLC accumulation, with 2 isolates having high FLC accumulation (B11789 and B12037), 2 isolates with low accumulation (B11215 and B11787), and 2 having intermediate levels of intracellular FLC (B11791 and B11801). This expanded isolate set also contained 4 resistant isolates (B11811, B11814, B11815, and B11895) that had high FLC MICs but also accumulated higher levels of FLC compared to other isolates with similar FLC MICs.

Of 119 C. auris isolates, 83 out of 87 resistant isolates (~95%) had extremely low fluconazole uptake, whereas 30 out of 32 susceptible isolates (~93%) had high fluconazole uptake. To determine the mechanism for this phenomenon, we first investigated the effect of the efflux transporter Cdr1 and the Tac1B transcription factor thought to regulate Cdr1 on FLC accumulation.

**Fig 3:**
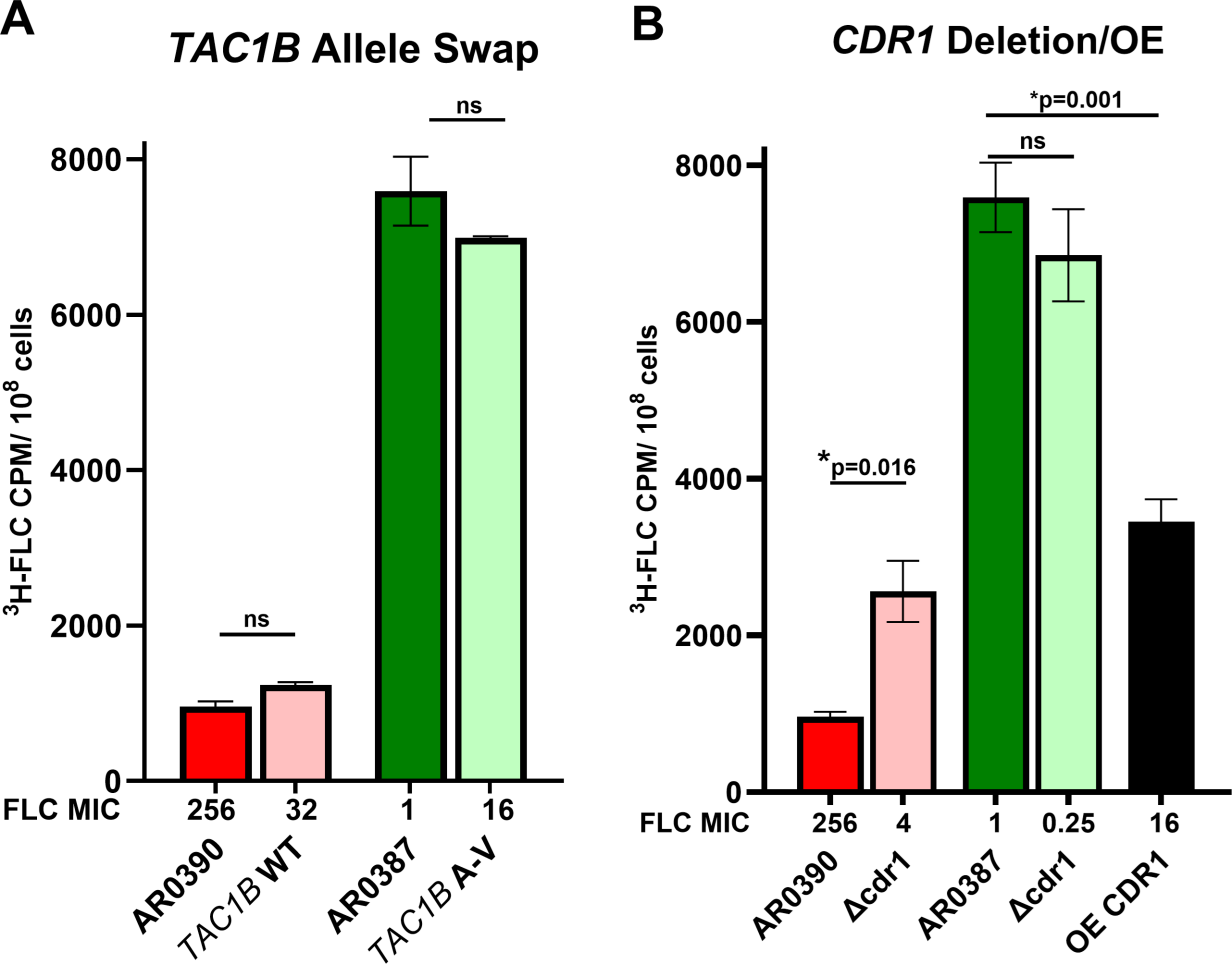
(**A**) TAC1B allele swap between FLC-resistant and -susceptible isolates. TAC1B swapping between strains altered the FLC MIC but did not significantly alter the FLC accumulation compared to their parent strain. (**B**) CDR1 deletion in FLC-resistant and -susceptible strains. CDR1 deletion (light red bar) in the resistant AR0390 parent (red bar) allowed significantly increased (*P* = 0.016) FLC accumulation, but not to the levels of a susceptible parent isolate (green bar). CDR1 deletion (light green bar) in the susceptible AR0387 parent did not significantly change FLC accumulation. The FLC MIC of each CDR1 deletion strain was reduced. CDR1 overexpression (black bar) in the susceptible AR0387 background strain significantly reduced (*P* = 0.001) FLC accumulation and increased the FLC MIC, but not to the level of resistant isolates.

We previously showed that mutations in the transcription factor TAC1B significantly contribute to fluconazole resistance when a susceptible isolate AR0387 (shown with high accumulation in Fig. 1) underwent rapid FLC-evolved resistance (20). All derived resistant strains in that study had TAC1B mutations and increased CDR1 expression (20). Figure 3A shows a further examination of the direct role/contributions of Tac1B in FLC accumulation. We measured FLC accumulation using TAC1B allele swap of parental strains AR0390 (FLC-resistant, low-accumulation, TAC1B mutant, shown in dark red bar), and strain AR0387 (FLC-susceptible, high-accumulation, TAC1B WT, shown in dark green bar). Figure 3A shows the results of FLC accumulation in these parental and allele-swap strains. When the WT TAC1B gene from the susceptible isolate was introduced into the FLC-resistant isolate, the MIC was reduced from 256 to 32 µg/mL (light red bar), and the FLC accumulation was not significantly changed compared to that of the parental isolate. FLC accumulation remained at a low level compared to the FLC-susceptible isolate from which the TAC1B allele was derived. When mutated TAC1B from the resistant isolate was introduced into the FLC-susceptible isolate, the MIC was increased from 1 to 16 µg/mL (light green bar); however, the FLC accumulation in this allele-swapped strain was not significantly changed.

Figure 3B shows examination of the role of Cdr1 in FLC accumulation. We next examined isogenic CDR1 knockout strains in which CDR1 was deleted in the resistant AR0390 (dark red bar) and susceptible AR0387 (dark green bar) parent isolates, as well as a CDR1 overexpression strain (black bar) in the AR0387 background (20). Figure 3B shows that the CDR1 deletion strains from both parent isolates had reduced FLC MICs, from 256 to 32 µg/mL in the AR0390 knockout strain (light red bar) and from 1 to 0.25 µg/mL in the AR0387 knockout strain (light green bar). There was significantly increased FLC accumulation in the AR0390 CDR1 KO strain (light red bar), but not to the levels of the AR0387-susceptible isolate. However, there was no significant change in FLC accumulation in the AR0387 CDR1 KO strain (light green bar) compared to the AR0387 parent. CDR1 overexpression increases the FLC MIC from 1 to 16 µg/mL (black bar) in the AR0387 background strain and significantly reduces FLC accumulation compared to the AR0387 parent strain, but FLC accumulation is not reduced to the level of the resistant isolates.

**Fig 4:**
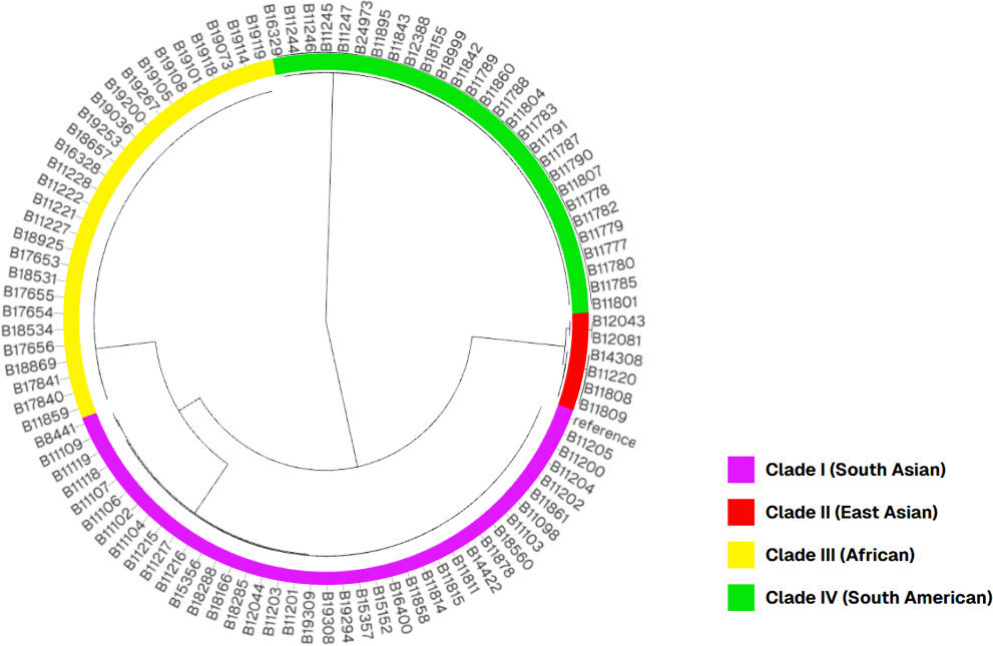
The neighbor-joining (NJ) phylogenetic tree of C. auris WGS used in this study. Inner colors represent the different C. auris clades. Branch lengths represent SNPs. Isolates clustered in the commonly known clades I–IV. The reference strain is B11205 (GCA-016772135.1). The NJ tree was visualized together with metadata containing additional data for each sample using Microreact (http://microreact.org).

To better understand the relatedness of C. auris isolates with high or low levels of intracellular FLC accumulation, we created a phylogenetic tree for most of the isolates in our collection based on whole-genome sequencing (WGS) and single-nucleotide polymorphisms (SNPs) (Fig. 4). There were no WGS data available for 10 of the isolates in our collection, and those were not included in the SNPs and phylogenetic analysis (B8441, B11098, B11105, B11244, B11245, B12037, B13285, B13286, B13287, and B19310), and those were left out of the tree. AR bank isolates shown in Fig. 1 are also not included in this SNP analysis.

**Fig 5:**
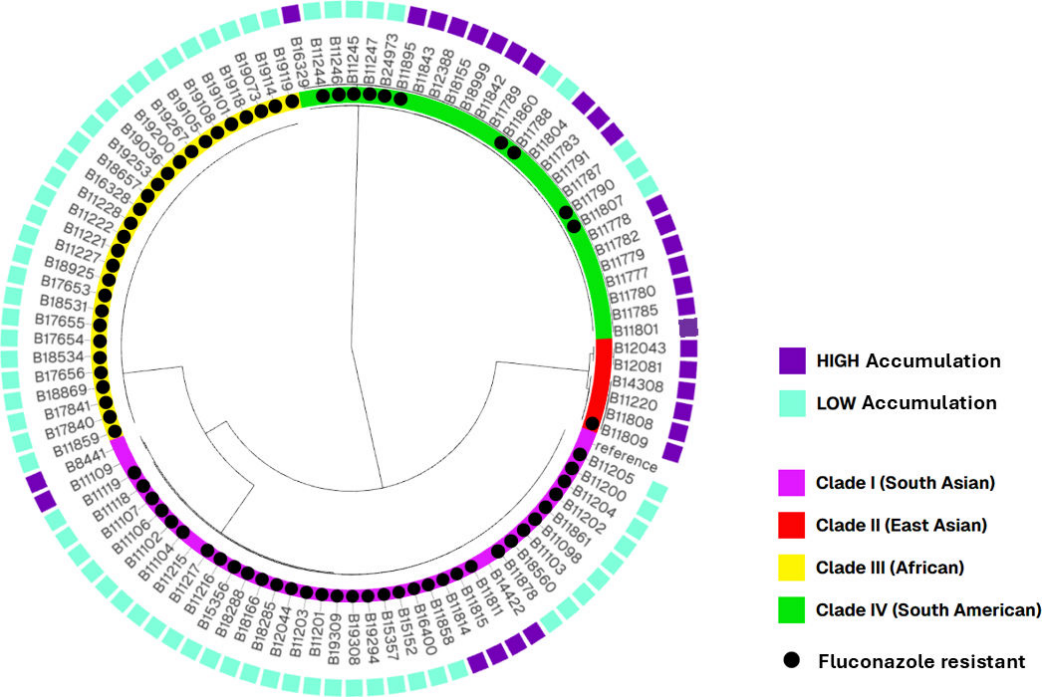
Overlap between FLC accumulation and FLC resistance. The NJ phylogenetic tree of C. auris WGS used in this study. Inner colors represent C. auris clades I–IV. Branch lengths represent SNPs. The reference strain is B11205 (GCA-016772135.1). Purple boxes indicate isolates with high FLC accumulation. Teal boxes indicate isolates with low FLC accumulation. Black circles indicate FLC-resistant isolates. The NJ tree was visualized together with metadata containing additional data for each sample using Microreact (http://microreact.org).

**Fig 6:**
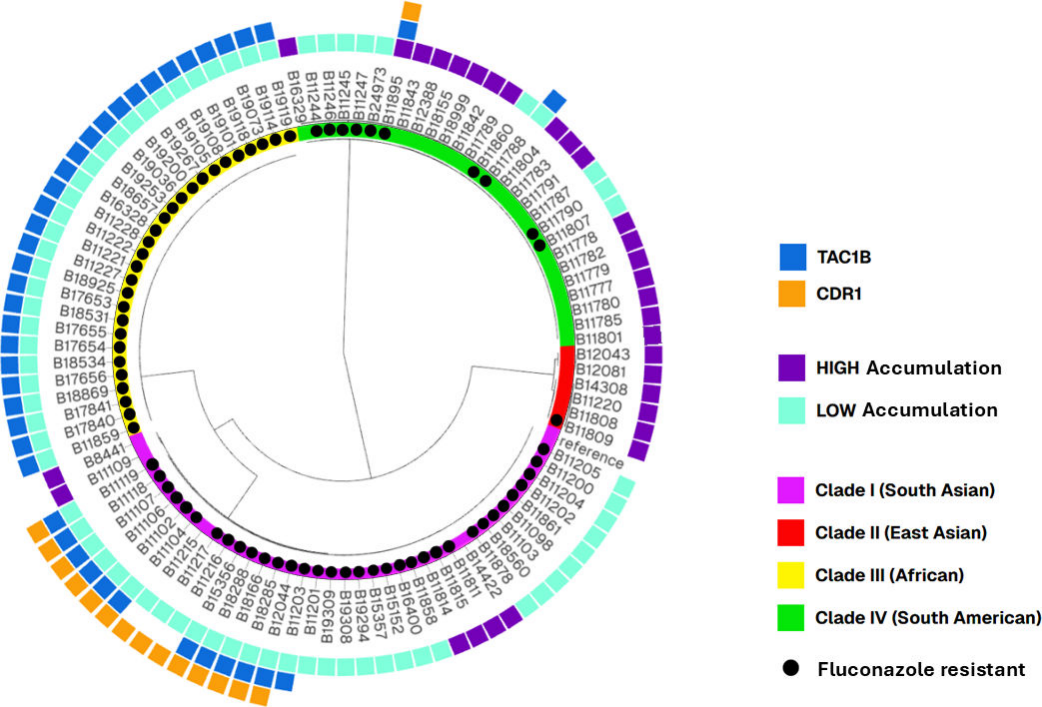
Genomic metadata overlap between genes related to efflux. The NJ phylogenetic tree of C. auris WGS used in this study. Inner colors represent C. auris clades I–IV. Branch lengths represent SNPs. The reference strain is B11205 (GCA-016772135.1). Purple boxes indicate isolates with high FLC accumulation. Teal boxes indicate isolates with low FLC accumulation. Black circles indicate FLC-resistant isolates. Blue boxes indicate isolates that contain one of the 7 common TAC1B resistance-associated mutations (583S, A15T/S195C, K247E, L582, P595H, R495G, and S89Y/E200K/K225N/I268V/Q298K/T346I/Q503R/F580L/Y647C). Orange boxes indicate isolates that contain one of the 2 common CDR1 resistance-associated mutations (E709D and E709G). The NJ tree was visualized together with metadata containing additional data for each sample using Microreact (http://microreact.org).

**Fig 7:**
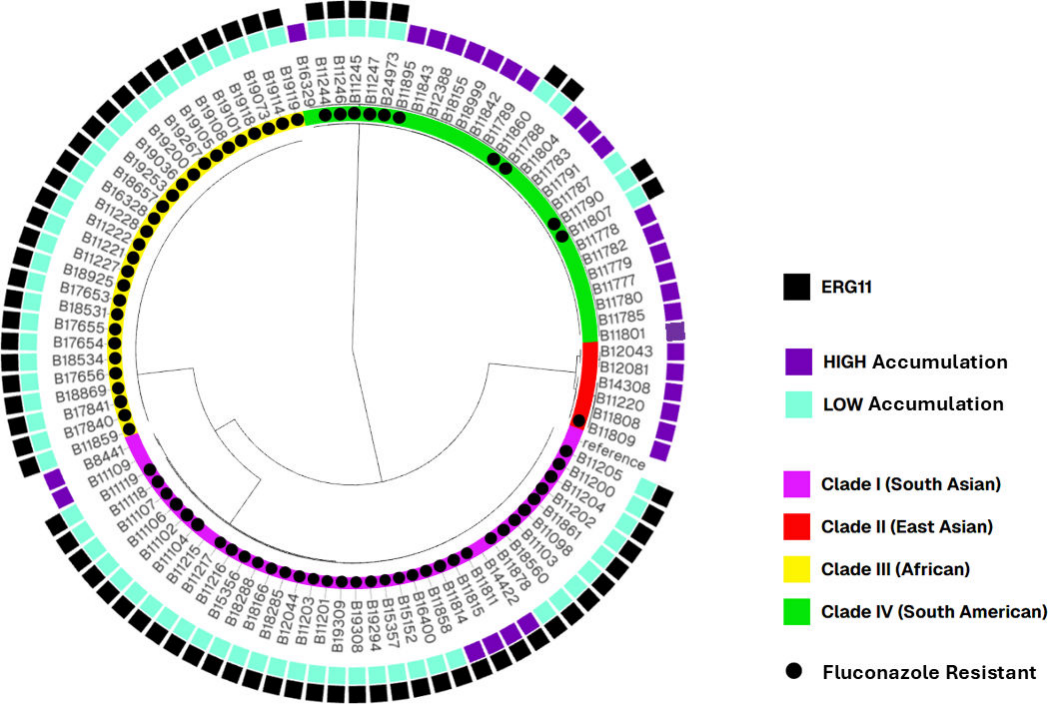
Genomic metadata overlap between FLC accumulation and ERG11 SNPs. The NJ phylogenetic tree of C. auris WGS used in this study. Inner colors represent C. auris clades I–IV. Branch lengths represent SNPs. The reference strain is B11205 (GCA-016772135.1). Purple boxes indicate isolates with high FLC accumulation. Teal boxes indicate isolates with low FLC accumulation. Black circles indicate FLC-resistant isolates. Black boxes indicate isolates that contain one of the 5 common ERG11 resistance-associated mutations (F126L, K143R, V125A/F126L, Y132F, and 7501H). The NJ tree was visualized together with metadata containing additional data for each sample using Microreact (http://microreact.org).

To identify a genetic mechanism that is specifically responsible for high or low levels of intracellular FLC accumulation, we searched for SNPs that would result in nonsynonymous polymorphisms in the genes TAC1B, CDR1, or ERG11 using SNPeff. SNPeff analysis showed that all isolates that were FLC-resistant showed at least one nonsynonymous polymorphism in the genes TAC1B, CDR1, or ERG11. The NJ tree was visualized together with metadata containing the SNPeff results using Microreact (Fig. 4 to 7; Sheet S1 and S2). Phylogenetic analysis showed that in general, isolates within each clade that showed the same nonsynonymous substitutions clustered together. This was observed for all three genes evaluated (TAC1B, CDR1, or ERG11).

Of the 100 C. auris WGS included in the SNP analysis, 73% (73/100) were resistant to FLC (MIC ≥32 µg/mL). Of those, 94% (69/73) also showed low FLC accumulation, while only 7% (2/27) susceptible isolates also showed low FLC accumulation, both exhibiting an elevated fluconazole MIC (16 ug/mL) just one dilution below the tentative breakpoint. Among the resistant isolates with low FLC accumulation (69), 25 showed one polymorphism in ERG11, two showed both CDR1 and ERG11 polymorphisms, 30 showed both ERG11 and TAC1B polymorphisms, and 11 showed polymorphisms in all three genes (CDR1, ERG11, and TAC1B). More analysis needs to be done to better understand the role of the gene polymorphisms associated with resistance and low intracellular FLC accumulation, although our results suggest that in general, multi-gene polymorphisms may be behind these mechanisms, including the involvement of ERG11 polymorphisms.

Unsurprisingly, the isolates clustered based on clade (Fig. 4), rather than FLC uptake, TAC1B, CDR1, or ERG11 genotype (Fig. 4 to 7). The metadata for TAC1B and CDR1 SNPs overlap with many but not all isolates that are FLC-resistant. CDR1 nonsynonymous polymorphisms were more common in resistant isolates from Clade I (Fig. 4 and 6; Sheet S1), and TAC1B mutations are commonly found in resistant isolates from clades I and III (Fig. 4 and 6; Sheet S1). There is clearly an association between FLC resistance and certain CDR1 and TAC1B polymorphisms, but these genes do not account for all mechanisms of resistance in this species and do not account for the difference in FLC accumulation.

When ERG11 SNPs were included in the analysis (Fig. 7; Sheet S1), there is a correlation between low FLC accumulation and isolates that have one of five ERG11 variants (Fig. 7). Conversely, there is a correlation between high FLC accumulation and isolates that have a non-variant ERG11 sequence. This ERG11/accumulation correlation is especially evident in Clade IV isolates. Figure 7 shows that for the isolates with SNP data, 69 out of 71 isolates with low FLC accumulation (97%) have a variant ERG11 sequence, including F126L, K143R, V125/F126L, Y132F, or Y501H. And 25 out of 29 isolates with high FLC accumulation (86%) have a non-variant ERG11 sequence. Variations in ERG11 could explain FLC susceptibility and differences in FLC accumulation. There is a cluster of related isolates that prove to be an exception to this rule: B11422, B11811, B11814, and B11815 have variant ERG11 but still have high FLC accumulation (Fig. 4 and 6; Sheet S1). Perhaps deeper genomic investigations or cell analysis could explain the high FLC accumulation phenomenon in this group. Additionally, as mentioned previously, a resistant isolate with high FLC accumulation (B11895) has non-variant ERG11, and two susceptible isolates with low FLC accumulation (B11215 and B11787) have variant ERG11 (Fig. 7; Sheet S1), which is consistent with Erg11 being a driver of FLC accumulation, separate from FLC susceptibility or resistance.

**Fig 8:**
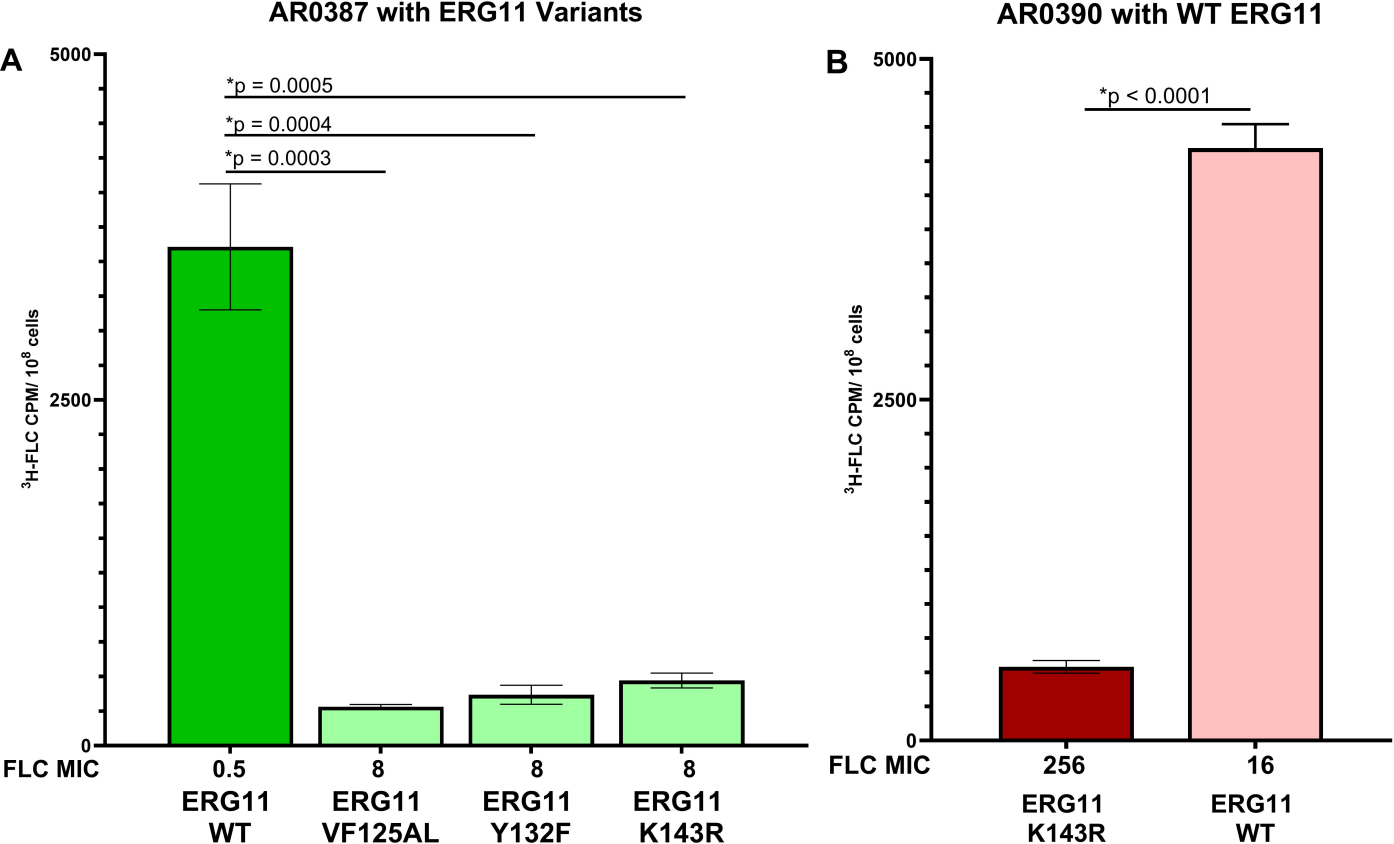
FLC accumulation in ERG11 allele swap strains. (**A**) FLC accumulation is significantly reduced (*P* ≤ 0.0005) when ERG11 variants VF125AL, Y132F, or K143R (light green) were introduced to the AR0387 parent (dark green). FLC MIC was increased with each of these allele swaps (8 µg/mL) compared to the susceptible parental strain (0.5 µg/mL). (**B**) FLC accumulation is significantly increased (*P* < 0.0001) when the non-variant ERG11 gene (light red) was introduced into the AR0390 parent (dark red). FLC MIC was reduced in this strain (16 µg/mL) compared to the resistant parent (256 µg/mL).

To illustrate the effect of the ERG11 sequence on FLC accumulation, we measured FLC accumulation in clinical isolates with ERG11 allele swaps (23). Figure 8 shows the susceptible isolate AR0387 expressing its native non-variant ERG11 (dark green bar), compared to the strain with each of the 3 common ERG11 variant sequences (light green bars) K143R, VF125AL, and Y132F. These ERG11 variant allele replacements increased the FLC MIC from 0.5 to 8 µg/mL, which is lower than that of the AR0390 parent strain (Clade 1) with the K143R variant (dark red bar). This suggests additional contributions to resistance apart from the ERG11 variant, such as Tac1B. FLC accumulation in these ERG11 variant allele-swap strains is significantly reduced (*P* ≤ 0.0005) compared to the parent isolate and is similar to the level of resistant isolates. Further, replacing the native variant K143R ERG11 sequence in the FLC-resistant parental isolate AR0390 (dark red bar) with a non-variant ERG11 sequence reduced the FLC MIC from 256 to 16 µg/mL (light red bar), which is still higher than that of the susceptible isolate (0.5 µg/mL). This non-variant ERG11 allele-swapped strain also had significantly increased (*P* < 0.0001) FLC accumulation compared to the resistant parent isolate and similar to the high level of accumulation observed in the susceptible isolate.

**Fig 9:**
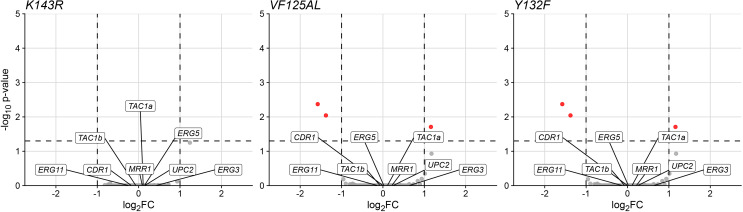
ERG11 resistance mutations do not lead to substantial changes in global transcriptional profiles. Volcano plots demonstrating differential expression between AR0387 mutants encoding each ERG11 resistance allele—K143R, VF125AL, or Y132F—compared to the parental non-variant strain. Genes that exhibited a log2-FC of 1 or greater and a *P*-value of 0.05 or less are highlighted in red. No genes were commonly dysregulated among the three mutant strains, and selected genes with known mechanisms of fluconazole resistance were not dysregulated in any strain, as indicated by labels.

To determine whether a transcriptional response to the mutation of ERG11 could contribute to the reduced uptake of fluconazole in each mutant, we performed RNA-Seq on each mutant strain to identify genes differentially expressed compared to the parental non-variant. We observed no evidence of substantial global dysregulation, with less than 1% of genes being significantly dysregulated in each mutant strain (Fig. 9; Sheet S3). Despite the phenotypic similarity in fluconazole uptake exhibited by the ERG11 K143R, VF125AL, and Y132F mutants, no genes were commonly dysregulated between these strains. We did not observe dysregulation of any genes that could plausibly be mechanistically connected to FLC resistance or uptake, and a selected set of known resistance genes showed no differential expression in any mutant (Fig. 9; Sheet S4). These data suggest that mutation of ERG11 toward any of the three resistance alleles does not lead to a substantial global transcriptional change and that a transcriptional shift is not likely to explain the reduced fluconazole uptake demonstrated by these strains.

## DISCUSSION

Of 119 C. auris isolates, 84 out of 87 resistant isolates (~95%) had extremely low fluconazole uptake, whereas 30 out of 32 susceptible isolates (~93%) had high fluconazole uptake. Fluconazole accumulation is affected by TAC1B mutations and CDR1 expression as illustrated by WT and mutated TAC1B allele swap and CDR1 deletion and overexpression strains (Fig. 3A and B). While these efflux-related genes are clearly a contributing factor to azole resistance in C. auris, they are not the exclusive determinant of drug uptake and accumulation in the C. auris cell. However, when we incorporated any of the three most common Erg11 variants into a strain (Y132F, K143R, and VF125AL), this was sufficient to reduce FLC accumulation to the very low levels seen in resistant isolates. Conversely, by incorporating a non-variant Erg11 into a resistant isolate, the derived strain had significantly increased FLC accumulation to the levels of susceptible isolates, and the MIC was reduced from 256 to 16 µg/mL. These results indicate that these ERG11 variations alone are necessary and sufficient to change the FLC accumulation phenotype.

Our molecular analyses provide additional support for the cell biology observations. When we overlay SNP variants with the drug resistance phenotype and FLC accumulation phenotype, the genes TAC1B/CDR1 are clearly correlated with resistance in some isolates (Fig. 6). But these efflux sequence variants are not found in all resistant isolates and do not explain the FLC accumulation phenotype. All isolates that contain a TAC1B resistance-associated mutation are FLC-resistant, and all but one isolate (B11895) has low FLC accumulation. However, in the randomly selected data set used in this study, there were many FLC-resistant isolates and/or isolates with low FLC accumulation that do not contain the TAC1B mutations (Fig. 6; Sheet S1, examples include B11204, B16400, B11790, B11860, and B11246), suggesting TAC1B mutations are sufficient but not necessary for FLC resistance. There are 15 isolates in our collection that contain a resistance-associated CDR1 mutation, and all but one (B11215) are FLC-resistant, and all but one (B11895) have low FLC accumulation (Fig. 6; Sheet S1). The majority of FLC-resistant isolates do not have the CDR1 mutations. Thus, resistance related to efflux has overlap with, but is a distinctive mechanism from, drug uptake. Conversely, when we overlap variants of ERG11 with the drug resistance phenotype and the FLC accumulation phenotype, there is a clear correlation between reduced drug accumulation and presence of ERG11 variants and increased drug accumulation in the absence of ERG11 variants, independent of Tac1/Cdr1 phenotypes (Fig. 6 and 7; Sheet S1). There is a particularly strong (1:1) correlation between the ERG11 variant and low drug accumulation in Clade IV. The isolate B11895 is an example that illustrates the importance of the ERG11 variants on drug uptake. This isolate is FLC-resistant, presumably via efflux-related resistance mechanisms from having both TAC1B and CDR1 mutations but still has high FLC accumulation and is the only drug-resistant isolate that does not have an ERG11 variant, further providing evidence that ERG11 plays a role in FLC accumulation, which is separate from Tac1-/Cdr1-related efflux. Additional examples include isolates B11215 and B11787, which are the only two FLC-susceptible isolates that have low FLC accumulation. Both of these isolates have a variant ERG11, which is correlated with the low FLC accumulation phenotype.

### Transporters

Drug resistance in human pathogens can sometimes be attributed to changes in their “transportome.” The entry of drugs into fungal cells has conventionally been thought to occur primarily via passive diffusion through the cell membrane. However, increasing evidence from our lab and others suggests that at least some antifungal drugs such as azoles are transported into cells by facilitated diffusion, although the identification of the responsible importer has not been determined. Drugs may enter cells using transporters whose physiological role is the import of biologically relevant molecules, such as hexose and other nutrients. In human parasites, decreased drug uptake due to drug-carrier alterations has been a well-characterized drug resistance mechanism (24), and similar hypotheses have been proposed for fungal drug import (25, 26).

Previous reports from our labs and others identified and demonstrated contributions of TAC1B and CDR1 for azole drug resistance in C. auris, consistent with efflux-associated resistance mechanisms in other fungal pathogens. In regard to C. auris efflux specifically, mutations in the transcription factor TAC1B and overexpression of CDR1 are associated with increased fluconazole resistance (19). In addition, there are only a small number of unique genes in C. auris that are absent in other pathogenic Candida species including C. albicans, C. glabrata, and the C. haemulonii clade (27). Some of these C. auris-specific genes encode ABC transporters, perhaps further contributing to its intrinsic antifungal-resistant nature (27).

With transporters in mind, to determine the mechanism for reduced fluconazole accumulation in azole-resistant C. auris isolates, we first investigated the effect of the efflux transporter Cdr1 and the Tac1B transcription factor, thought to regulate Cdr1, on FLC accumulation (Fig. 3). When a WT TAC1B sequence replaces a mutated TAC1B in a resistant isolate, the FLC MIC is reduced from 256 to 32 µg/mL, and FLC uptake is relatively unchanged and remains substantially below the levels of uptake observed in the susceptible parental isolate. When a TAC1B sequence containing resistance-associated mutations (A640V) replaces a WT TAC1B in a susceptible isolate, there is reduced (but not statistically significant) FLC uptake in these cells; however, the MIC is increased (Fig. 3A). These results demonstrate again that mutations in Tac1B in C. auris are associated with elevated fluconazole MICs but have lesser contributions to the reduction in FLC accumulation in the resistant isolates. There is some other factor influencing drug entry into the cell.

When CDR1 is deleted or disrupted in FLC-resistant isolates, the KO/derived strains have increased FLC uptake, but not to the levels of a susceptible isolate (Fig. 3B). When CDR1 is overexpressed in an FLC-susceptible isolate, FLC accumulation is reduced significantly, but not to the low level of the resistant isolates. Disruptions to CDR1 reduce FLC MIC in all KO strains, contribute to reduced intracellular FLC accumulation in C. auris, and are clearly important mechanisms of resistance in this species; this is not the exclusive determining factor for FLC accumulation.

Our data show that initial FLC accumulation or uptake into the cell is variable between isolates and an independent phenomenon from efflux from the cells, illustrating separate processes and machinery involved in the drug entering the cell and getting the drug back out of the cell. We found that FLC accumulation level can be predicted based on FLC MIC and vice versa and is caused by some additional contributing factors in the strains apart from Tac1B and Cdr1.

### Erg11 and ergosterol

Fluconazole inhibits cellular ergosterol biosynthesis by targeting ERG11 that is essential for the production of ergosterol (8). Thus, many fungal adaptations to fluconazole treatment involve changes to the expression of ERG11 or the conformation of the enzyme. The ERG11 allele swap showed that each of these three singular ERG11 variants (Y132F, K143R, and F126L or VF125AL) is sufficient to change the FLC accumulation phenotype (Fig. 7).

The Y132F mutation has been described and associated with reduced azole susceptibility in isolates of C. auris, C. albicans, C. orthopsilosis, C. parapsilosis, C. tropicalis, and A. fumigatus (19, 28–30). Beyond the effect of reduced azole binding to the enzyme, point mutations in ERG11 can also contribute to higher levels of ergosterol during FLC treatment and, surprisingly, even yield overexpression of ERG11. One example is the point mutation K143R located in the exposed active site of the Erg11 enzyme. The K143R amino acid substitution in Erg11 causes alterations in the tertiary structure of Erg11, resulting in a lower binding affinity of azoles to this enzyme (23). This K143R mutation has been found in fluconazole-resistant C. albicans and C. auris isolates, as well as pan-azole resistant isolates of C. tropicalis (19, 23, 31). In a C. albicans isolate, the K143R mutation increased ERG11 expression, resulting in high levels of ergosterol and reduced fluidity in the plasma membrane. Additionally, the C. albicans K143R mutation resulted in significant cell wall remodeling, facilitated by changes to the cell-surface chitin and β-glucan (32).

Increased ERG11 gene expression has previously been associated with the upregulation of the transcription factor UPC2. In the case of ERG11 hot spot/active site mutations, it is unlikely that UPC2 has an influence on the increased expression as UPC2 binds the ERG11 promoter sequence, leaving the upregulation mechanism unknown. Nevertheless, synonymous codon substitutions in ERG11 have also been shown to lead to the increased expression of the ERG11 gene in itraconazole-resistant C. krusei isolates. In this species, the A756T mutation in Erg11 resulted in upregulation of ERG11 and a major ABC transporter Abc2, leading to itraconazole resistance. itraconazole accumulation was significantly reduced in the ITC-resistant C. krusei isolates, similar to the fluconazole accumulation pattern we see in C. auris (33). In this analysis of C. krusei, the difference in drug accumulation was proposed to be due to a change in membrane permeability as the sterol/phospholipid ratio in the lipid bilayer was altered, with contributions of the enhanced efflux of the antifungal agent. These examples demonstrate that point mutations in the ERG11 gene can have a pleiotropic effect on the cell processes and morphology. However, our RNA-seq data did not show significant gene expression changes in the ERG11 allele swap strains (Fig. 9; Sheet S3 and S4).

**Fig 10:**
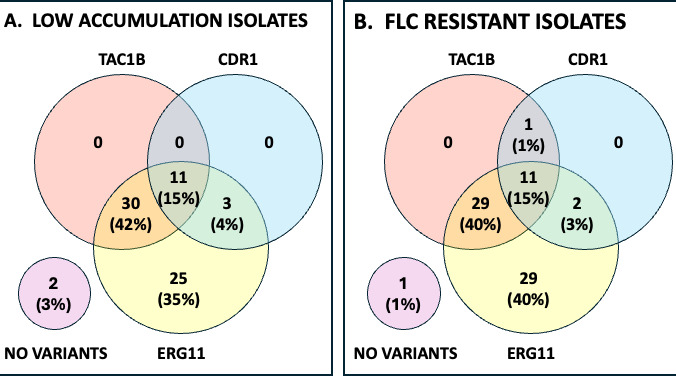
Gene contributions to accumulation and resistance in C. auris isolates. (**A**) A total of 71 isolates with low FLC accumulation are evaluated for the presence of TAC1B, CDR1, and ERG11 gene variants. (**B**) A total of 73 isolates with FLC resistance are evaluated for the presence of the same variants. The genome sequence of other isolates in the collection was not available.

A summary of the variants in ERG11, CDR1, and TAC1B is presented in Fig. 10 for 71 strains with lowered FLC accumulation in the cell and for 73 strains with FLC resistance. Figure 10 shows that the majority of isolates with low FLC accumulation and the majority of isolates that are FLC resistant have variant ERG11. Only two FLC-resistant isolates are devoid of ERG11 variants—one has no detectable variants in resistance genes, and one has both TAC1B and CDR1 variants. Only one isolate, with no detectable variants, is associated with low FLC accumulation.

### Complex system of interactions

The dynamic relationship between the ergosterol pathway and drug transport may converge in the fungal membrane composition. Mutations in the ERG11 sequence can lead to a complexity of cellular effects related to ERG11 expression, sterol regulation, and plasma membrane composition. Preliminary sterol analysis of the ERG11 mutants did not identify any changes in ergosterol levels or shifts in sterol content (data not shown). Changes to sterol content or incorporation of modified sterols might alter membrane fluidity, which could allow more or fewer molecules to pass through in both directions (8). Lipid rafts are membrane regions with significant quantities of embedded lipids, sterols, and proteins (34). These highly specialized domains compartmentalize cellular functions. For example, S. cerevisiae’s important transporters Pdr10, Aus1p, and Pdr11p are associated with lipid raft domains (35, 36). It has been shown that the treatment of S. cerevisiae with FLC corresponded to the reduction of plasma membrane rigidity, leading to decreased order of lipids in the membrane (35). This suggests that the altered sterol composition after azole treatment impacts lipid organization too, resulting in decreased fluidity of the membrane.

Additionally, the activity of ABC transporters is influenced by the presence or absence of certain membrane components so that membrane homeostasis and physical structure can potentially result in altered transporter localization and function (34, 36). Conversely, mislocalization of transporters can alter the other components of the membrane, leading to incorrect organization and dysfunction. These effects could alter drug import and efflux; for example, the reduction of ergosterol or sphingolipids has been shown to affect membrane localization of a major multidrug exporter protein Cdr1p in C. albicans (37). Also, deletion of the plasma membrane transporter gene CDR6 and subsequent removal of the protein from the membrane caused increased plasma membrane rigidity in C. albicans, resulting in reduced azole accumulation and contributing to azole resistance (38). These studies highlight the complex interactions between membranes and transport and the far-reaching, pleiotropic effects that mutations in the ERG11 gene could induce.

## MATERIALS AND METHODS

### Isolates, strains, and growth media used in this study

Strains used in this study are listed in Table 2. The clinical C. auris isolates AR0381, AR0382, AR0383, AR0384, AR0385, AR0386, AR0387, AR0388, AR0389, and AR0390 were obtained from the CDC and FDA Antimicrobial Resistance (AR) Isolate Bank collection of C. auris isolates (22). Isolates DI17-46, DI17-47, and DI17-48 were obtained from Dr. Dave Rogers, St. Jude Children’s Research Hospital, Memphis, TN. All but one C. auris B-numbered isolates were obtained from the CDC. Isolate B12037, also known as LSPQ-01061, was obtained from Leah Cowen with permission from Philippe Dufresne of Laboratoire de santé publique du Québec. All clinical isolates and constructed strains were stored as frozen stocks in 50% glycerol at −80°C. All clinical isolates and strains were routinely grown in Complete Supplemental Mixture (CSM) broth (0.79 g complete supplemental powder, 1.7 g yeast nitrogen base [YNB], 5 g ammonium sulfate, and 20 g dextrose per liter) in a shaking incubator at 35°C unless otherwise indicated for specific experimental conditions.

**TABLE 2:** Strains used in this study

Strain ID	#	Received from	Reference
AR0381-390	10	Antifungal Resistance Isolate Bank	This paper
DI17-46-48	3	Dave Rogers, St. Jude, TN	This paper
B8441, B11098, and B11102–B19310	105	CDC-Shawn Lockhart	This paper
B12037 (LSPQ-01061)	1	Philippe Dufresne via Leah Cowen	This paper
AR0387 Δcdr1	1	Dave Rogers, St. Jude, TN	(39)
AR0390 Δcdr1	1	Dave Rogers, St. Jude, TN	(39)
AR0387 TAC1B A640V	1	Dave Rogers, St. Jude, TN	(40)
AR0390 TAC1B WT	1	Dave Rogers, St. Jude, TN	(40)
AR0387 ERG11 VF125AL	1	Dave Rogers, St. Jude, TN	(23)
AR0387 ERG11 Y132F	1	Dave Rogers, St. Jude, TN	(23)
AR0387 K143R	1	Dave Rogers, St. Jude, TN	(23)
AR0390 ERG11 K143R	1	Dave Rogers, St. Jude, TN	(23)
AR0390 ERG11 NON-VARIANT	1	Dave Rogers, St. Jude, TN	(23)

### Assessment of [^3^H]-fluconazole accumulation

C. auris parental isolates and all derived strains were grown overnight in liquid CSM at 35°C shaking. Cells were pelleted and washed 3× with YNB and then glucose-starved for 3 h. Concentrated cell pellets (50 µL) were added to 400 µL of PBS (pH 7) with or without glucose and 50 µL of freshly diluted 0.77 µM [^3^H]-fluconazole. This yielded a final [^3^H]-fluconazole concentration of 23.6 pg/L (+/− 2% glucose), which is significantly below the MIC of each strain or isolate being tested. Samples were then incubated, with shaking at 30°C for 24 h, and the OD600 nm for each sample was recorded. Heat-killed samples (100°C for 15 min) were used as controls for nonspecific drug binding and background signals. Stop solution (5 mL) (PBS +20 mM [6 mg/L] unlabeled fluconazole) was added to each sample in a 14 mL round-bottom tube. Samples were filtered on glass fiber filters and then washed with another 5 mL of PBS before the filters containing cells were then transferred to a 5 mL scintillation vial. A Beckman Coulter LS6500 scintillation analyzer (Beckman Coulter, USA) was then used to quantify the radioactivity of each filter in 3 mL of scintillation cocktail (Ecoscint XR, National Diagnostics) as Counts Per Minute (CPM) over the course of 1 min. Experiments were performed in duplicate with biological replicates, and all results were normalized to CPM per 1 × 10^8^ cells.

### Fluconazole susceptibility testing

MICs for fluconazole (Fisher Scientific) were determined by broth microdilution in 96-well plates in accordance with the M27-A4 standard from the Clinical and Laboratory Standards Institute (41) with minor adjustments. Each isolate was grown in Complete Supplemental Mixture (CSM) with 2% glucose in 96-well plates containing a gradient of fluconazole in a 2-fold serial dilution starting at 256 µg/mL and incubated at 35°C with shaking for 24 h. MIC plates’ absorbance was measured at 600 nm on a BioTek 96-well plate reader. All MIC analyses were performed in technical triplicate, and the values were averaged.

### Statistical analysis

All [^3^H]-fluconazole accumulation experiments were performed in triplicate or higher replicates, and error bars represent standard errors. Unpaired *t*-tests were used to compare the means of the different groups to determine if there was a statistically significant difference between them. Statistical significance is presented as a *P*-value and considered significant if *P* < 0.05.

### TAC1B and ERG11 allele swap strains

Allele swap strains were previously created and characterized (23, 40). For the TAC1B allele swaps, TAC1B WT came from Clade 1 isolate AR0387, and TAC1B A640V came from Clade 1 isolate AR0390. For the ERG11 allele swaps, WT ERG11 came from Clade 1 isolate AR0387, K143R ERG11 came from Clade 1 isolate AR0390, VF125AL ERG11 came from Clade 3 isolate AR0383, and Y132F ERG11 came from Clade 4 isolate DI-19-24.

### SNP and phylogenetic analysis

WGS of the 100+ C. auris isolates was conducted at the U.S. CDC, Mycotic Diseases Branch (MDB), previously described (2). SNPs were identified using MycoSNP v1.4 as described by Bagal et al. (42). Analyses were conducted using C. auris strain B11205 [GenBank Accession no. GCA-016772135.1]. Genetic distance calculations and NJ tree construction were performed using MEGA 11. The MycoSNP pipeline generated the VCFs from the filtered SNP calling file. Using the VCFs and SnpEff (v 5.1) (43), we searched for SNPs in the TAC1B, CDR1, or ERG11 gene ortholog (gene IDs: CAB11_004683, CAB11_002795, and CAB11_003031 for the C. auris reference genome GCA-016772135.1) in all analyzed genomes. All nonsynonymous polymorphisms in the respective genes were retrieved. The N41 tree was visualized together with metadata containing additional epidemiologic data and nonsynonymous polymorphisms found in the genes for each sample using Microreact (http://microreact.org).

### RNA-seq

In triplicate, RNA was extracted from the ERG11 allele swap strains, grown to the mid-log phase in YPD at 35°C using a formamide extraction method, as previously described (44). RNA sequencing and WGS analysis were performed as previously described (40). Reads were aligned to the parental AR0387 WT strain (also known as B8441_v2 assembly) (GCA_002759435.2), and differential expression between each strain harboring mutant ERG11 alleles and the parental non-variant strain was assessed using limma-voom. During differential expression calculations, lowly expressed genes were filtered at a cutoff of 0.5 cpm, and *P*-values were adjusted using a Benjamini and Hochberg correction. Significance was determined relative to a log2FC of 1 and a *P*-value of 0.05.

## SUPPLEMENTAL MATERIAL

Supplemental Sheets mbio.03957-25-s0001.xlsx Sheets S1-S4.

ASM does not own the copyrights to Supplemental Material that may be linked to, or accessed through, an article. The authors have granted ASM a non-exclusive, world-wide license to publish the Supplemental Material files. Please contact the corresponding author directly for reuse.
